# Identification of fatty acid metabolism-related lncRNAs in the prognosis and immune microenvironment of colon adenocarcinoma

**DOI:** 10.1186/s13062-022-00332-y

**Published:** 2022-07-28

**Authors:** Shuang Wu, Yuzhu Gong, Jianfang Chen, Xiang Zhao, Huimin Qing, Yan Dong, Sisi Li, Jianjun Li, Zhe Wang

**Affiliations:** 1 Department of Oncology and Southwest Cancer Center, Southwest Hospital, Third Military Medical University (Army Medical University), Chongqing, 400038 China; 2 Key Laboratory of Tumor Immunopathology of Ministry of Education of China, Institute of Pathology and Southwest Cancer Center, Southwest Hospital, Third Military Medical University (Army Medical University), Chongqing, 400038 China

**Keywords:** Colon adenocarcinoma, Fatty acid metabolism, Long non-coding RNAs, Molecular subtypes, Prognosis, Immune infiltration

## Abstract

**Background:**

Cancer metabolism is largely altered compared to normal cells. This study aims to explore critical metabolism pathways in colon adenocarcinoma (COAD), and reveal the possible mechanism of their role in cancer progression.

**Methods:**

Expression data and sequencing data of COAD samples were obtained from The Cancer Genome Atlas and Gene Expression Omnibus databases. The expression profiles between tumor and normal samples were compared to identify differential metabolism pathways through single sample gene set enrichment analysis.

**Results:**

Fatty acid synthesis was identified as a key metabolism pathway in COAD. Based on fatty acid-related lncRNAs, two molecular subtypes (C1 and C2) were defined. C2 subtype with worse prognosis had higher immune infiltration and higher expression of immune checkpoints. Five transcription factors (TFs) including FOS, JUN, HIF1A, STAT3 and STAT2 were highly expressed in C2 subtype. Five fatty acid-related lncRNAs were identified to be biomarkers for predicting COAD prognosis. Finally, further experients showed that knockdown of lncRNA PAXIP1-AS1 decreased the triglyceride content and the fatty acid synthase and acetyl-CoA carboxylase 1 expressions, which suggested that lncRNA PAXIP1-AS1 plays an important role in fatty acid metabolism of COAD.

**Conclusions:**

This study demonstrated that fatty acid synthesis was greatly altered in COAD. Fatty acid-related lncRNAs were speculated to be involved in cancer progression through associating with TFs. The five screened TFs may serve as new drug targets for treating COAD.

**Supplementary Information:**

The online version contains supplementary material available at 10.1186/s13062-022-00332-y.

## Introduction

Colon adenocarcinoma (COAD) is one of the most common cancers within digestive system, whose death rate ranking in the fifth of all cancer types [1]. American Joint Committee on Cancer (AJCC) classifies COAD patients into different stages with different clinical features including histological features, metastasis and lymph node. With the development of new therapies for cancer treatment, especially immunotherapy and other molecular-targeted therapies, improved overall survival of cancer patients is shown [2, 3]. However, not all cancer patients can benefit from these new therapeutics. To address this issue, a number of studies also explored a series of molecular features such as genomic features [4] and biomarkers [5–7] for predicting the response to immunotherapy or chemotherapy, thus guiding personalized treatments.

Cancer metabolism is one of important aspects in cancer development, such as activated glycolysis contributing a lot in cancer metastasis [8]. The complexity of cancer metabolism has been underscoring in the previous studies, but comprehensive understanding on cancer metabolism can largely facilitate the development of new targeted drugs. ACLY is the first-step rate-controlling enzyme in lipid synthesis, which is found to be upregulated in colon cancer and to contribute metastasis [9]. Evidences have demonstrated that the remodeling of tumor microenvironment (TME) can be modulated by metabolism-related pathways [10]. Conversely, TME can also alter metabolic features through cellular metabolites of cells in TME [11]. It has been suggested that targeting cancer metabolism is a promising strategy for cancer treatment [12–15]. Lipid metabolism-related signature is also developed for predict colon cancer survival [16].

In the regulation of metabolism-related pathways, long non-coding RNAs (lncRNAs) play a critical role in metabolic reprogramming [17–19]. For example, lncRNA-GLCC1 was found to reprogram glycolytic metabolism and was associated with prognosis in colorectal cancer [20]. CCAT1/FABP5 promotes tumour progression through mediating fatty acid metabolism in lung adenocarcinoma [21]. LINC01606 protects colon cancer cells from ferroptotic cell death and functions as an oncogene to facilitate tumor cell stemness [22]. CDKN2B-AS1 is considered as a promising biomarker or therapeutic target in various cancer types [23].

Therefore, in this study, we introduced a series of bioinformatics analysis to further explore the role of metabolism and metabolism-related lncRNAs in COAD development. We identified fatty acid biosynthesis as a critical pathway that was greatly dysregulated in COAD. Based on lncRNAs related to fatty acid, we constructed two novel molecular subtypes with different molecular features. Furthermore, we explored the relation between lncRNAs and transcription factors (TFs), and identified key fatty acid-related lncRNAs for predicting COAD prognosis. Fatty acid was considered as a critical metabolism pathway in COAD progression.

## Methods

Additional file 1: Fig. S1A shows the workflow of this study.

### Information of COAD samples and data preprocessing

TCGA-COAD dataset containing RNA-seq data (FPKM) and clinical information was downloaded from The Cancer Genome Atlas (TCGA) database in October 01, 2021. GSE17538 dataset was obtained from Gene Expression Omnibus (GEO) database in October 01, 2021. Samples without clinical information, survival time and survival status were removed. Only samples with stage II, III and IV were remained. Ensembl ID and probes were converted to gene symbol. The fpkm of gene expression was converted into TPM expression profile according to the method of Li et al. [24]. Expression data in two datasets were identified into mRNAs and lncRNAs according to GTF file downloaded from GENCODE (https://www.gencodegenes.org/).

### Identification of fatty acid-related lncRNAs

Pearson correlation analysis was conducted between fatty acid activity score and lncRNAs. Fatty acid-related lncRNAs were identified under conditions of |correlation coefficient|> 0.25 and *P* < 0.05. The lncRNAs that were identified both in TCGA-COAD and GSE17538 datasets were considered as important fatty acid-related lncRNAs for further analysis.

### Unsupervised consensus clustering based on fatty acid-related lncRNAs

Unsupervised consensus clustering is a widely used method for identifying molecular subtypes with different molecular features based on expression profiles. We used ConsensusClusterPlus R package to conduct unsupervised consensus clustering based on the expression of fatty acid-related lncRNAs [25]. KM algorithm was used and Euclidean was selected as distance. 500 bootstraps were repeated with each bootstrap containing 80% samples in TCGA-COAD dataset. Cluster number k was set from 2 to 10, and cumulative distribution function (CDF) and consensus matrix were used to confirm the optimal cluster number.

### Gene set enrichment analysis (GSEA)

GSEA is a powerful method for delineating biological processes based on gene expression data [26]. We applied GSEA to calculate the enrichment score of hallmark pathways for molecular subtypes. Hallmark pathways with a series of gene sets were obtained from Molecular Signature database (MSigDB) [27]. ClusterProfiler R package was used to annotate gene ontology (GO) terms and Kyoto Encyclopedia of Genes and Genomes (KEGG) pathways on fatty acid-related lncRNAs and TFs [28].

### Characterization of tumor microenvironment

The proportion of different immune cells was calculated by GSEA based on a series of gene signatures. Estimation of STromal and Immune cells in MAlignant Tumours using Expression data (ESTIMATE) algorithm was conducted to evaluate immune infiltration from two aspects including stromal score and immune score [29]. ESTIMATE score is the combined score of stromal score and immune score. Tumor Immune Dysfunction and Exclusion (TIDE) analysis was implemented for assess T cell function [30].

### Assessing the function of fatty acid-related lncRNAs

To analyze the localization of fatty acid-related lncRNAs, relative concentration index (RCI) was calculated for each lncRNA based on LncATLAS database [31]. RCI < 0 indicates nucleus-localized lncRNAs and RCI < 0 indicates cytoplasm-localized lncRNAs. The TF activity was assessed in accordance with the algorithm from Garcia-Alonso et al. [32]. Pearson correlation analysis was conducted to analyze the association between lncRNAs and TFs.

### The first-order partial correlation analysis

The first order partial correlation analysis was an efficient method for analyzing one variable in the relation between another two variables. It was used to investigate the association among fatty acid-related lncRNAs, fatty acid score and fatty acid-related genes [33]. The association between two variables was largely weakened when removing the effect of another variable. Of the case, the variable was considered as key fatty acid-related lncRNA.

### Construction of a prognostic model based on key fatty acid-related lncRNAs

Key fatty acid-related lncRNAs were identified by the first order partial correlation analysis. Univariate Cox regression analysis was employed on the key lncRNAs to obtain coefficients. The prognostic model was defined as risk score = beta i * expression i, where beta represents coefficients and i represents lncRNAs. Risk score was calculated for each sample in two datasets. Median value of risk score was selected as a cut-off to divide samples into high-risk and low-risk groups.

### Cell culture

NCM460 cell line (normal human colonic epithelial cell line) and all the four human colorectal cancer (COAD) cell lines (LoVo, HCT116, SW480, and HT-29) were purchased from the Cell Bank of Type Culture Collection of the Chinese Academy of Sciences (CBTCCCAS, Shanghai, China). The cells were cultured in RPMI 1640 medium (Gibco, United States) or McCoy’s 5A medium (Gibco, United States) supplemented with 10% fetal bovine serum (Gibco, United States), 100 U/ml penicillin, and 100 mg/ml streptomycin at 37 ℃ in a humidified incubator with a 5% CO2 concentration.

### Quantitative real-time PCR

Total RNA was extracted from NCM460 cells and COAD cells by TRIzol Reagent (Beijing Solarbio Technology Co., Ltd., Beijing, China) according on the manufacturer’s instruction. PrimeScript™ RT-PCR kit (TaKaRa, Mountain View, CA) was used to perform reverse transcription after quality validation. qRT-PCR was conducted by using the SYBR Premix Ex Taq™ (TaKaRa). The expression of β-actin was used as an internal control. The fatty acid-related lncRNA expression was measured via the 2^−ΔΔCT^ method. All primer sequences used in this research are listed in Additional file 2: Table S1.

### Short interference RNAs (siRNAs), and transfection

Small interfering RNA (siRNA) of PAXIP1-AS1 were synthesized by RiboBio (Guangzhou, China). HCT116 and HT-29 cells were transfected with si-PAXIP1-AS1for 48 h by using Lipofectamine 2000 (Invitrogen, CA, USA). qRT-PCR was used to evaluate the knockdown efficiency of si-PAXIP1-AS1.

### Intracellular triglyceride assay

Intracellular triglyceride was determined using an E1687Ge General TGC/Triglyceride ELISA Kit (EIAab Science Inc., Wuhan, China) at 450 nm according to the manufacturer’s protocol.

### Western blot

HCT116 and HT-29 cells transfected with si-PAXIP1-AS1 were lysed with RIPA lysis buffer (R0010, Solarbio, China) supplemented with protease inhibitors (Roche). BCA Kit (Pierce, Rockford, IL) was used to determine the protein concentration. The total protein was added into loading buffer and then was separated by 10% sodium dodecyl sulfate polyacrylamide gel electrophoresis (SDS-PAGE), then the separated protein was transferred to polyvinylidene fluoride (PVDF) membrane (Merck Millipore, Billerica, MA). The membrane was blocked with 5% skimmed milk for 1 h, the primary anti-fatty acid synthase (FASN) and acetyl-CoA carboxylase 1 (ACC1) antibodies (Cell Signaling Technology, Danvers, MA) was used to block protein overnight at 4 °C. The PVDF membrane was then washed with TBST and incubated with the corresponding secondary antibody for 2 h. The proteins were identified by using Pierce SuperSignal West Pico Chemiluminescent Substrate (Termo Fisher, Waltham, MA) according to the manufacturer’s protocol. β-actin antibody was used as the internal reference.

### Statistical analysis

Parameters of packages and tools were default if without indication. Statistical analysis was performed in R (4.1.1) platform. Statistical methods were indicated in the corresponding sections. *P* < 0.05 was considered as significant. ns, no sifnificance. *P < 0.05, **P < 0.01, ***P < 0.001, ****P < 0.0001.

## Results

### Identification of important metabolism-related pathways

Firstly, single sample gene set enrichment analysis (ssGSEA) was used to calculate enrichment score for each sample in two datasets. Then Wilcoxon test was conducted to identify differential metabolism-related pathways between normal and tumor samples in TCGA-COAD dataset. Within the identified differential pathways, univariate Cox regression analysis was performed to identify metabolism-related pathways that were associated with prognosis in two datasets. Finally, fatty acid biosynthesis pathway was identified in both two datasets.

### Constructing molecular subtypes based on lncRNAs related to fatty acid

**Fig. 1:**
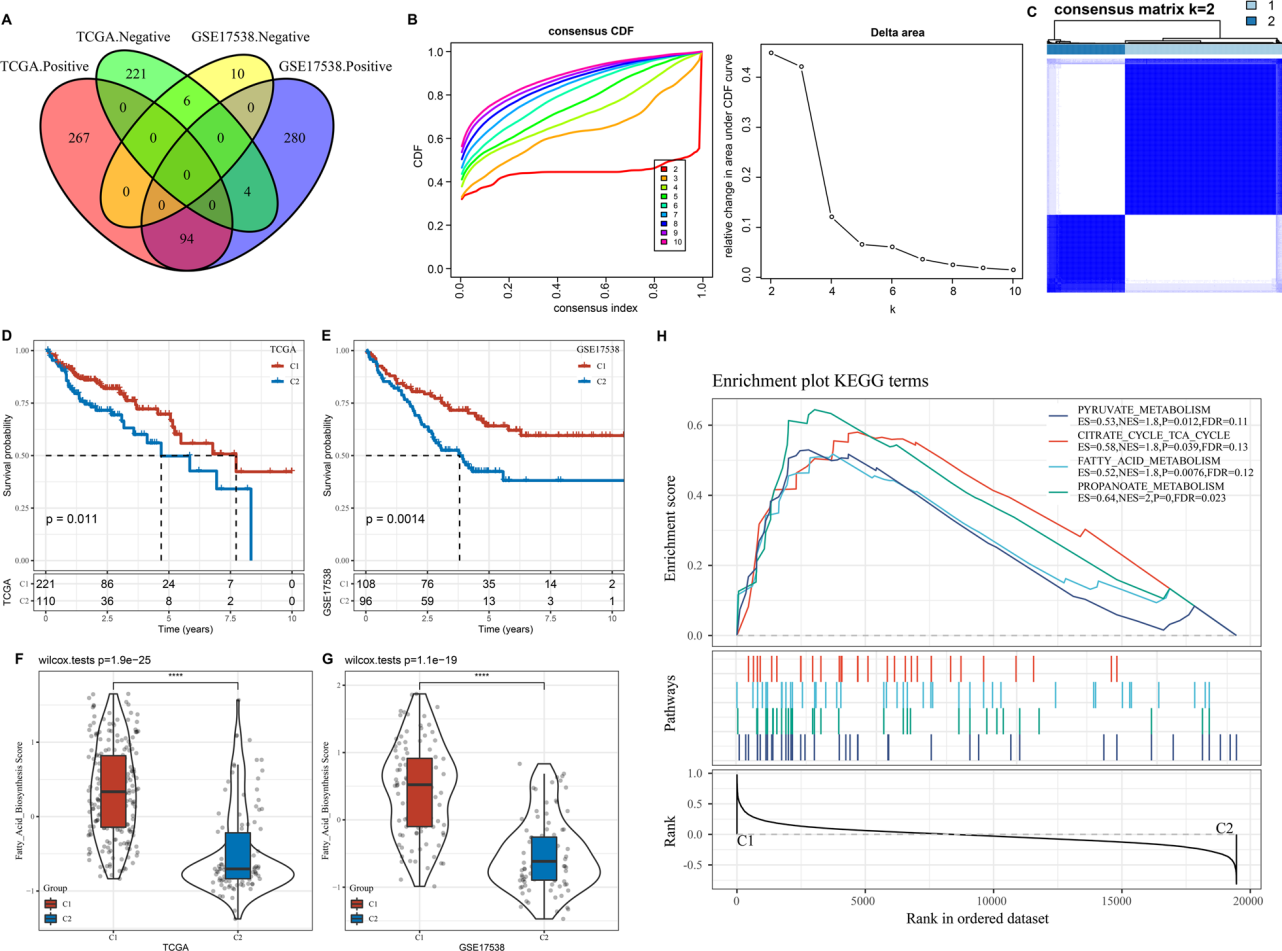
Identification of two molecular subtypes based on fatty acid-related genes. **A** Venn plot of fatty acid-related lncRNAs between TCGA-COAD and GSE17538 datasets. Positive indicates upregulated and negative indicates downregulated lncRNAs. **B** CDF curve and delta area of CDF curve when cluster number k = 2–10. **C** Consensus matrix when k = 2. **D, E** Kaplan–Meier survival curve of C1 and C2 subtypes in TCGA-COAD (**D**) and GSE17538 (**E**) datasets. Log-rank test was conducted. **F, G** Fatty acid activity score of C1 and C2 subtypes in TCGA-COAD (**F**) and GSE17538 (**G**) datasets. Wilcoxon test was performed. **H** GSEA on KEGG pathways from MSigDB for two subtypes in TCGA-COAD dataset

To identify lncRNAs related to fatty acid, Pearson correlation analysis was performed between activity score of fatty acid and lncRNA expression. In TCGA-COAD and GSE17538 datasets, we identified 592 and 394 lncRNAs related to fatty acid respectively. Among these lncRNAs, 6 lncRNAs were negatively correlated with fatty acid in both two datasets, and 94 lncRNAs were positivity correlated with fatty acid (Fig. 1A). Then univariate Cox regression analysis was conducted within 100 lncRNAs, and 15 lncRNAs related to prognosis were identified. By using unsupervised consensus clustering, samples in TCGA-COAD dataset were classified into different groups. According to CDF and area under CDF curve in different cluster numbers, cluster number k = 2 was determined to clearly classify samples into two groups (Fig. 1B, C). As a consequence, cluster 1 (C1) and cluster 2 (C2) were defined as two molecular subtypes. Survival analysis on two subtypes showed that C1 and C2 had differential overall survival with C1 had more favorable prognosis in both TCGA-COAD and GSE17538 datasets (*P* = 0.011 and *P* = 0.0014 respectively, Fig. 1D, E). Moreover, C1 subtype had extremely higher fatty acid activity than C2 subtype in both TCGA-COAD and GSE17538 datasets (*P* = 1.9e-25 and *P* = 1.1e-19 respectively, Fig. 1F, G), indicating that high fatty acid activity may have a protective role to against tumor progression. To understand if there was a difference on pathways between C1 and C2 subtypes, we used GSEA to calculate enrichment score of hallmark gene sets from MSigDB [27]. As a result, we observed that metabolism-related pathways including pyruvate metabolism, fatty acid metabolism, and citrate cycle TCA cycle were significantly enriched in C1 subtype (*P* < 0.05, FDR < 0.25, Fig. 1H).

### Different genomic and mutation features between C1 and C2 subtypes

**Fig. 2:**
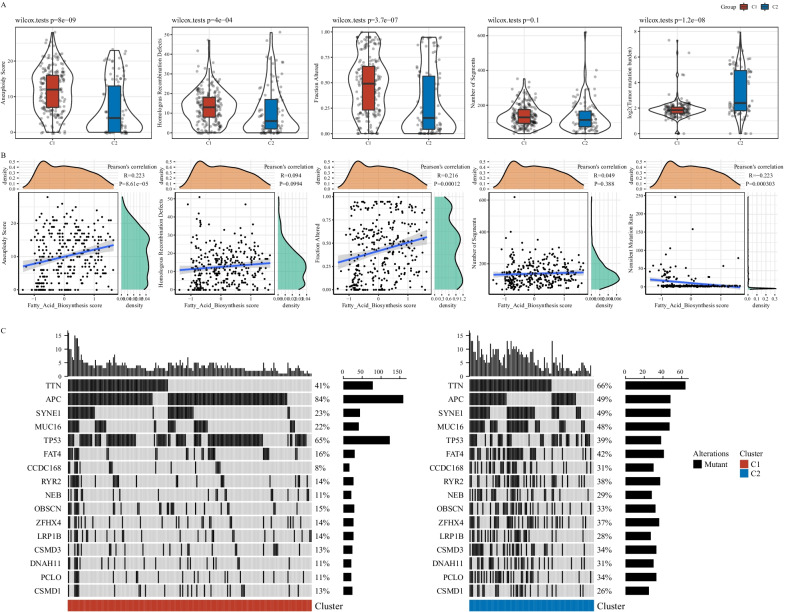
Genomic features and gene mutations of two subtypes. **A** The score of five genomic features including aneuploidy, homologous recombination defects, fraction altered, number of segments and tumor mutation burden. Wilcoxon test was performed. **B** Pearson correlation analysis between genomic features and fatty acid activity. **C** The top 16 significantly mutated genes in TCGA-COAD dataset. Fisher test was performed. Horizontal axis indicates samples and vertical axis indicates genes

To compare genomic features of two subtypes, we obtained genomic data of TCGA-COAD dataset from previous research [34], including aneuploidy score, homologous recombination defects, number of segments, fraction altered and tumor mutation burden. Except for number of segments, C1 and C2 subtypes had a significant difference on other four genomic features (*P* < 0.0001, Fig. 2A). C1 subtype showed obviously higher enrichment score of aneuploidy, homologous recombination defects and fraction altered, while C2 subtype had higher score of tumor mutation burden. Among five genomic features, aneuploidy score and fraction altered were significantly correlated with fatty acid score (R = 0.223, *P* = 8.61e-05 and R = 0.216, *P* = 0.00012 respectively, Fig. 2B). Furthermore, analysis on single nucleotide variations revealed the top 16 significantly mutated genes (*P* < 0.05, Fig. 2C). In C1 subtype, APC and TP53 were highly mutated with mutation frequencies of 84% and 65% respectively, while they were 49% and 39% in C2 subtype. Compared with C1 subtypes, mutations in C2 subtype were more scattered. The different genomic features and gene mutations can alter gene expression and thus contribute to dysregulated pathways.

### Differentially enriched pathways between C1 and C2 subtypes

**Fig. 3:**
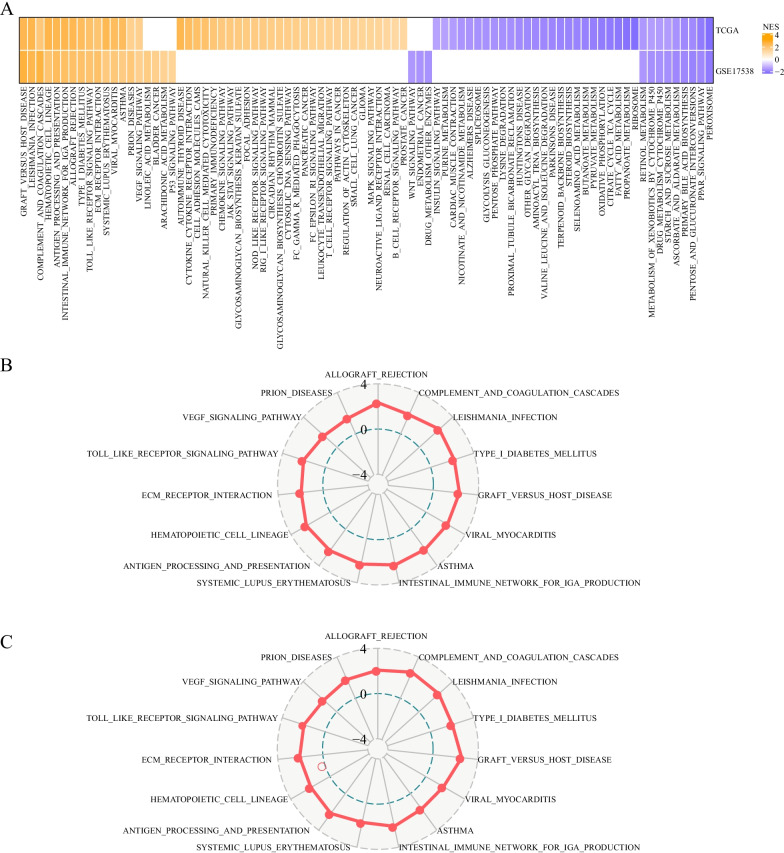
The enrichment of hallmark pathways in two subtypes. **A** Differential enriched pathways between C1 and C2 subtypes. NES was shown by comparing C2 with C1. Orange indicates relatively activated pathways and purple indicates relatively suppressed pathways in C2 subtype. **B, C** Rader plots of 15 pathways that were activated in C2 subtype in both TCGA-COAD (**B**) and GSE17538 (**C**) datasets. NES was shown between -4 to 4 from inner to outside circles

Given that C2 subtype had worse prognosis than C1 subtype, we tried to find out whether there were differentially enriched pathways that may affect prognosis between them. For this purpose, hallmark pathways from MSigDB were included for conducting GSEA in TCGA-COAD and GSE17538 datasets. By comparing C2 to C1, we observed that two subtypes exhibited differential enrichment in a number of pathways, with C2 subtype showing lower enrichment of many metabolism-related pathways. Within these differential pathways between two subtypes, 15 pathways such as VEGF signaling, ECM receptor interaction and Toll-like receptor signaling pathways were upregulated in C2 subtype in both two datasets, and 9 pathways such as retinol metabolism, P450, starch and sucrose metabolism were downregulated (Fig. 3A). Within 15 upregulated pathways in C2 subtype, Rader plots presented similar normalized enrichment score (NES) in two datasets (Fig. 3B, C), suggesting that the subtyping based on fatty acid-related lncRNA expression was reliable, and the these lncRNAs may be involved in regulating these activated pathways in C2 subtype.

### High immune infiltration and T cell dysfunction were shown in C2 subtype

**Fig. 4:**
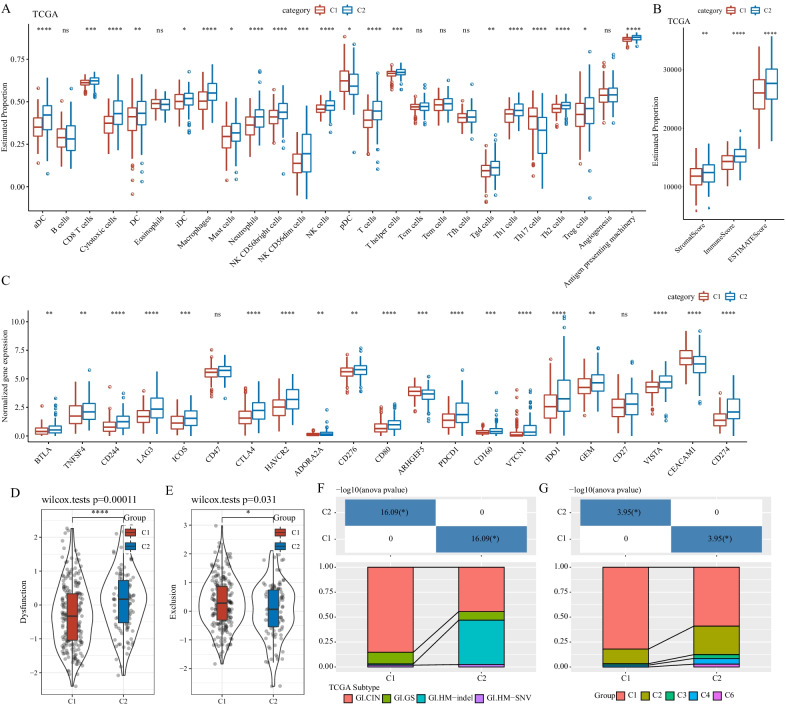
TME features of C1 and C2 subtypes in TCGA-COAD dataset. **A** Estimated proportions of 24 immune cells. **B** Stromal score, immune score and ESTIMATE score calculated by ESTIMTAE analysis. **C** The expression of immune checkpoints in two subtypes. **D, E** T cell dysfunction and exclusion score calculated by TIDE analysis. Wilcoxon test was conducted. **F** The intersection of the molecular subtypes in this study with the four previously reported molecular subtypes. **G** The intersection of the molecular subtypes of this study with the six previously reported immune molecular subtypes

The component of TME is a critical hallmark in cancer development. Therefore, we evaluate the distribution of different types of immune cells in two subtypes by using a series of gene signatures. As a result, among 24 immune cells, we found that 19 of them were differentially enriched in two subtypes in TCGA-COAD dataset (Fig. 4A). Notably, CD8 T cells, cytotoxic cells and dendritic cells (DCs) were more enriched in C2 subtype, while immunosuppressive cells such as macrophages and regulatory T (Treg) cells were also highly distributed (*P* < 0.05). ESTIMATE analysis further supported the above result that C2 subtype had significantly higher stromal score and immune score than C1 subtype, indicating higher immune infiltration of C2 subtype (*P* < 0.01, Fig. 4B). In GSE17538 dataset, we observed similar results (Additional file 1: Fig. S1A, B).

Furthermore, we investigated the expression of immune checkpoints that can affect the function of anti-tumor immune cells. It was observed that most of immune checkpoints were more highly expressed in C2 subtype than C1 (Fig. 4C). Especially, some important checkpoints whose high expression were reported to inhibit T cell function were significantly higher expressed in C2 subtype, such as LAG3, CTLA4, PDCD1, IDO1, and CD274 (*P* < 0.0001). High expression of these immunosuppressive checkpoints in C2 subtype may explain its worse outcome. In addition, TIDE analysis further demonstrated that C2 subtype had more severe T cell dysfunction than C1 (*P* = 0.00011, Fig. 4D), which was consistent with the above results. Simultaneously, T cell exclusion was shown to be a bit more severe in C1 (*P* = 0.031, Fig. 4E). In GSE17538 dataset, the expression of immune checkpoints were not obviously differential between two subtypes, but CD274 and CTLA4 were still overexpressed in C2 subtype (*P* < 0.01, Additional file 1: Fig. S1C). TIDE analysis in GSE17538 dataset displayed similar result with the result in TCGA-COAD dataset (Additional file 1: Fig. S1D and E), supporting that the unfavorable prognosis of C2 subtype may result from the dysfunction of cytotoxic T cells. We also compared the relationship between the two molecular subtypes and the four molecular subtypes previously reported by Liu et al. [35]. It can be observed that there are significant differences in the distribution of the corresponding four old molecular subtypes in the two subtypes, and the C1 subtype is mainly GI CIN subtype and C2 subtype are rich in GI Patients with HM-indel subtype (Fig. 4F). Thorsson et al. [34] reported the 6 immune subtypes in pan cancers. We compared these 6 subtypes with our C1 and C2 subtypes. Similarly, we can observe that there are significant differences in the distribution of 6 different immune subtypes in C1 and C2 subtypes in this study (Fig. 4G). Moreover, in the modulation of TME, fatty acid-related lncRNAs played a nonnegligible role.

### Fatty acid-related lncRNAs were involved in regulating oncogenic pathways

**Fig. 5:**
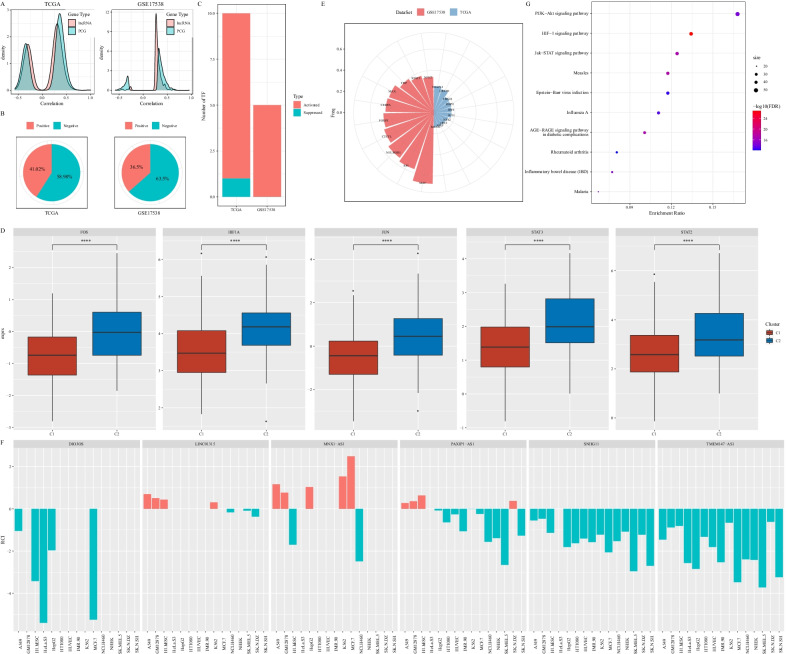
The interaction between fatty acid-related lncRNAs and TFs. **A** Pearson correlation analysis between fatty acid-related lncRNAs and PCGs. Horizontal axis represents correlation coefficient. **B** RCI distribution in two datasets. RCI > 0 indicates localization in the nucleus. RCI < 0 indicates localization in the cytoplasma. **C** Dysregulated TFs including activated and suppressed in two datasets. **D** The expression of 5 upregulated fatty acid-related lncRNAs in TCGA-COAD dataset. Student t test was conducted. **E** Fatty acid-related lncRNAs that were negatively correlated with dysregulated TFs in two datasets. **F** Six fatty acid-related lncRNAs identified to be significantly correlated with TF activity. **G** The top 10 enriched KEGG pathways of 5 upregulated fatty acid-related lncRNAs in TCGA-COAD dataset. Size indicates gene counts

Given that two molecular subtypes based on fatty acid-related lncRNAs displayed distinct molecular features and prognosis, we suspected that these lncRNAs may be highly involved in delivering tumor-promotive signals in COAD. LncRNAs are considered as important regulators in controlling gene expression. No surprisingly, we observed that the expression of protein-coding genes (PCGs) was highly consistent with the expression of fatty acid-related lncRNAs, with a higher proportion of positive correlations (Fig. 5A). To understand the localization of fatty acid-related lncRNAs which largely decided their function, LncATLAS database was utilized for calculating their RCI [31]. The result showed that fatty acid-related lncRNAs were localized more in the nucleus (RCI < 0) in both TCGA-COAD and GSE17538 datasets, with a proportion of 58.98% and 63.50% respectively (Fig. 5B).

LncRNAs regulating gene expression through suppressing or activating transcription factors (TFs) is one of the main mechanism. Next we analyzed TF activity of C1 and C2 subtypes according to the algorithm developed by Garcia-Alonso et al. [32], and identified 108 and 76 dysregulated TFs in TCGA-COAD and GSE17538 datasets respectively. We found that these dysregulated TFs were mostly activated (Fig. 5C), and five TFs were found to be upregulated in C2 subtype in both two datasets including FOS, HIF1A, JUN, STAT3 and STAT2 (*P* < 0.0001, Fig. 5D). Based on Pearson correlation analysis between these dysregulated TFs and nucleus-localized fatty acid-related lncRNAs, a group of TFs negatively associated with the lncRNAs were identified (*P* < 0.05, Fig. 5E). Within 100 fatty acid-related lncRNAs, 6 lncRNAs in the nucleus were identified to be significantly associated with TF activity, including DIO3OS, LINC01315, MNX1-AS1, PAXIP1-AS1, SNHG11, and TMEM147-AS1 (Fig. 5F). We inferred that these 6 lncRNAs may be greatly involved in regulating activated TFs observed in C2 subtype. Moreover, to better understand the function of the 5 upregulated TFs in C2 subtype, KEGG pathways were annotated. Consequently, three tumor-related pathways were identified within the top 10 significantly enriched pathways, including PI3K-Akt signaling, HIF-1 signaling and JAK-STAT signaling pathways (Fig. 5G).

### Identification of key fatty acid-related lncRNAs

**Fig. 6:**
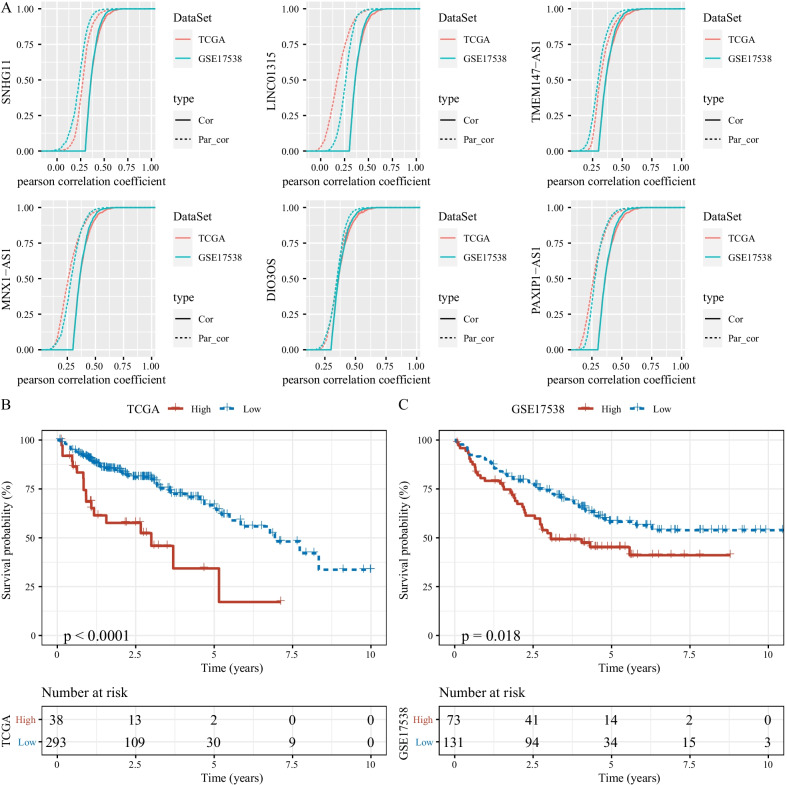
Identification of key fatty acid-related lncRNAs. **A** The first order partial correlation analysis for the 6 fatty acid-related lncRNAs. Solid lines and dashed lines indicate the correlation coefficients between fatty acid-related genes and fatty acid activity before and after removing the effect of fatty acid-related lncRNAs respectively. **B, C** Kaplan–Meier survival analysis of high-risk and low-risk groups in TCGA-COAD (**B**) and GSE17538 (**C**) datasets. Log-rank test was conducted

To identify key lncRNAs that were involved in regulating fatty acid metabolism, we applied the first order partial correlation analysis among fatty acid-related genes, fatty acid activity and the identified 6 fatty acid-related lncRNAs that were associated with TF activity. As a consequence, 5 fatty acid-related lncRNAs were screened in two datasets (Fig. 6A). The correlation between fatty acid activity and fatty acid-related genes was significantly weakened when removing these fatty acid-related lncRNAs, indicating that these lncRNAs played important roles in the signals related to fatty acid. Functional analysis on these 5 lncRNAs revealed that metabolism-related pathways such as purine metabolism, pyrimidine metabolism and other glycan degradation were enriched (Additional file 3: Fig. S2). Based on the expression of 5 lncRNAs, high-risk and low-risk groups were defined according to the Optimal cut-off of risk score. Two groups manifested distinct overall survival in both two datasets (Fig. 6B, C), indicating that these 5 lncRNAs could serve as biomarkers to predict prognosis for COAD patients.

### Effects of lncRNA PAXIP1-AS1 on fatty acid metabolism of colorectal cancer

**Fig. 7:**
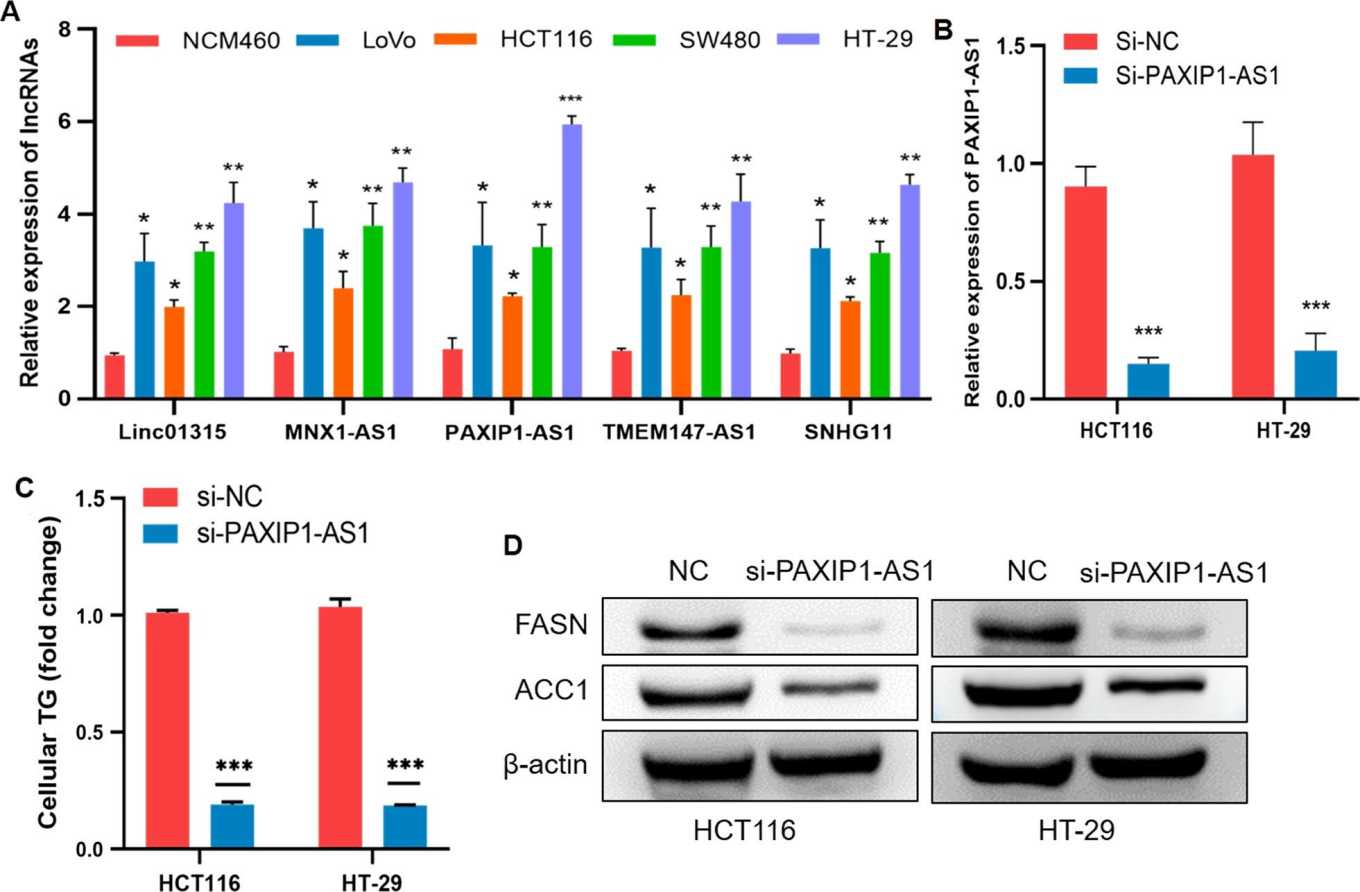
Effects of lncRNA PAXIP1-AS1 on fatty acid metabolism in COAD cells. **A** qRT-PCR was used to determine the mRNA expression of five lncRNAs in the normal human colonic epithelial cell line NCM460 and 4 COAD cell lines. **B** Knockdown efficiency of the HT-29 and HCT116 cell lines transfected with si-PAXIP1-AS1was confirmed by qRT-PCR. **C** The effect of si-PAXIP1-AS1 on the triglyceride content in COAD cells. **D** FASN and ACC1 protein expression in COAD cells were examined by western blots. Data are shown as mean ± SD, *P < 0.05, **P < 0.01, ***P < 0.001

To furthermore explore the role of lncRNAs on fatty acid metabolism of colorectal cancer, we determined the expression of five lncRNAs (linc01315, MNX1-AS1, PAXIP1-AS1, SNHG11, TMEM147-AS1) in 4 human COAD cell lines (LoVo, HCT116, SW480, and HT-29) and the normal human colonic epithelial cell line NCM460, the results showed that compared with NCM460 cell line, the mRNA expression levels of five lncRNAs in the 4 COAD cell lines were all up-regulated, of which, HT-29 cell line exhibited the highest expression of five lncRNAs, while HCT116 cell line exhibited the lowest expression of five lncRNAs (Fig. 7A). Therefore, HT-29 and HCT116 cell lines were chosen as candidate cell lines for the following experiment. Among the five lncRNAs, lncRNA PAXIP1-AS1 showed the highest expression, then we explored the role of PAXIP1-AS1 on fatty acid metabolism in COAD cells. qRT-PCR showed the expression of PAXIP1-AS1 were significantly decreased in the si-PAXIP1-AS1 HT-29 and HCT116 cell lines (Fig. 7B). Afterward, intracellular triglyceride content was determined by the triglyceride assay kit, the result showed that knockdown of PAXIP1-AS1 reduced the triglyceride content in COAD cells (Fig. 7C). Consistently, FASN and ACC1, the key enzymes of fatty acid synthesis metabolism, were significantly downregulated in si-PAXIP1-AS1 COAD cells (Fig. 7D). Together, these results suggest that lncRNA PAXIP1-AS1 accelerates the fatty acid metabolism of COAD.

## Discussion

In the comparison of metabolism-related pathways between normal and tumor samples, we identified that fatty acid synthesis pathway was significantly dysregulated in COAD samples. Then based on the expression of lncRNAs related to fatty acid, we constructed two molecular subtypes that had differential prognosis. C2 subtype had unfavorable prognosis and lower the enrichment of fatty acid synthesis, compared with C1 subtype. High activity score of fatty acid seemed to play a protective role in COAD.

Fatty acid synthesis is an essential part of lipid metabolism, which can convert nutrients into metabolic components for synthesizing biological membranes, storing energy and generating signaling molecules [36]. Fatty acid synthesis has been demonstrated to be involved in tumorigenesis and tumor progression in various cancer types. We found that some tumor-related pathways such as VEGF signaling, Toll-like receptor signaling and ECM receptor signaling were associated with high activity of fatty acid synthesis. VEGF is a key mediator of angiogenesis in cancer, which can promote metastasis by activating VEGF signaling. In colorectal cancer, fatty acid was observed to be higher expressed in VEGF-positive tumor tissues, compared to VEGF-negative tumor tissues [37], which was accordant with our study. Inhibiting fatty acid synthase can suppress angiogenesis through modulating VEGF-A expression in glioma cells [38]. Zaytseva et al. also support that knock-down of fatty acid synthase decreases the expression of VEGF and thus suppress angiogenesis in colorectal cancer [39].

Toll-like receptors (TLRs) contributes to activate immune response in inflammation-induced diseases and cancers, which is stimulated by saturated fatty acids [40, 41]. Overexpression of TLR4 indicates progression and poor prognosis in colon cancer [42]. The previous findings suggest that high expression of fatty acid can promote cancer progression through activating VEGF signaling and Toll-like receptor signaling, however, which seems inconsistent to our observation that C1 subtype had higher activity of fatty acid synthesis and higher enrichment of these pathways.

We put our sight into TME, one of critical hallmarks of cancer, which determines the prognosis to some extent. C2 subtype had significantly higher immune infiltration but also higher expression of immune checkpoints than C1 subtype, which may result in its worse prognosis. Especially, high expression of LAG3, CTLA4, PDCD1 (PD-1), IDO1, and CD274 (PD-L1) were widely reported to be associated with suppressive immune response and suppressed T cell function in cancer. Immune checkpoint blockade such as anti-CTLA-4 and anti-PD-1/PD-L1 have been considered as promising strategies for treating various cancer types also including COAD [43].

An interaction between fatty acid metabolism and TME has been reported in a number of studies. In glioblastoma, Tregs obtain energy relying on fatty acid oxidation (FAO) under hypoxic conditions [44]. Within the process, hypoxia-inducible factor 1α (HIF-1α) is an inducer to drive Tregs depending on lipid oxidation in mitochondrial metabolism. M2 macrophages, which are associated with cancer progression and invasion secret increased IL-1β under conditions of FAO dependence and HIF-1α upregulation [45]. Therefore, fatty acid metabolism plays an important role in TME alternation.

To further reveal the mechanism of fatty acid metabolism in cancer development, the association between fatty acid-related lncRNAs and TFs were analyzed. We identified six fatty acid-related lncRNAs that had close association with dysregulated TFs, including DIO3OS, LINC01315, MNX1-AS1, PAXIP1-AS1, SNHG11, and TMEM147-AS1. Furthermore, five TFs were found to be significantly upregulated in C2 subtype in both two datasets, including FOS, HIF1A, JUN, STAT3 and STAT2. HIF1A gene expressing HIF-1α contributes to hypoxia-induced FAO dependence, which is consistent with the previous findings [44, 45]. STAT3 and STAT2 belong to STAT family and are involved in STAT signaling, which are highly associated with TME and cancer progression [46]. Especially, STAT3 signaling induces the upregulation of immunosuppressive factors such as TGF-β and PD-1/PD-L1, and promotes angiogenesis through upregulating HIF-1α and VEGF [47]. Higher expression of STAT2 and STAT3 may lead to the worse prognosis of C2 subtype. However, the relation between fatty acid-related lncRNAs and these TFs except for HIF1A remains to be unclear, and it needs exploring in the further study.

To validate the function of the lncRNAs in the fatty acid metabolism-related signature we constructed, lncRNA PAXIP1-AS1 was selected to conduct functional experiments in COAD cells. The results showed that knockdown of PAXIP1-AS1 decreased the content of triglyceride in COAD cells. As we know, FASN and ACC1were the key enzymes involving in the fatty acid synthesis metabolism, both of them modulate the lipogenesis, growth, and apoptosis of COAD cells [39, 48]. Besides, the upregulation of FASN is correlated with the metastasis in COAD [49, 50]. We then explored the expression of FASN and ACC1 in si-PAXIP1-AS1 COAD cells, the result showed the expression of FASN and ACC1 were decreased when PAXIP1-AS1 expression was downregulated. Taken together, this study demonstrates that PAXIP1-AS1 plays a key role in the fatty acid metabolism of COAD.

## Conclusions

In conclusion, based on comparing the molecular features between C1 and C2 subtypes, we considered that fatty acid-related lncRNAs were involved in modulating fatty acid synthesis and TME, and thus contributes to cancer progression. A possible relation was identified between fatty acid-related lncRNAs and immunosuppressive factors such as HIF-1α and STAT. The five identified TFs may be new targets for exploiting new molecular drugs. Moreover, five fatty acid-related lncRNAs were screened to establish a prognostic signature for predicting COAD prognosis.

## Supplementary Information

**Additional file 1.** **Supplementary Fig. S1:** Workflow and TME features of C1 and C2 subtypes in GSE17538 dataset. **A** Work flow chart. **B** Estimated proportions of 24 immune cells. **C** Stromal score, immune score and ESTIMATE score calculated by ESTIMTAE analysis. **D** The expression of immune checkpoints in two subtypes. **E, F **T cell dysfunction and exclusion score calculated by TIDE analysis. Wilcoxon test was conducted.

**Additional file 2.** **Supplementary Table S1:** Primer Sequences used in qRT-PCR.

**Additional file 3.** **Supplementary Fig. S2:** Functional analysis of 5 key fatty acid-related lncRNAs.

## Data Availability

TCGA-COAD dataset containing RNA-seq data (FPKM) and clinical information was downloaded from The Cancer Genome Atlas (TCGA) database in October 01, 2021. GSE17538 dataset was obtained from Gene Expression Omnibus (GEO) database in October 01, 2021.
