# Supplementary figures and images for: Identification of fatty acid metabolism-related lncRNAs in the prognosis and immune microenvironment of colon adenocarcinoma
Source: Biol Direct. 2022 Jul 28;17:19. doi: 10.1186/s13062-022-00332-y (PMC9331591; doi:10.1186/s13062-022-00332-y)

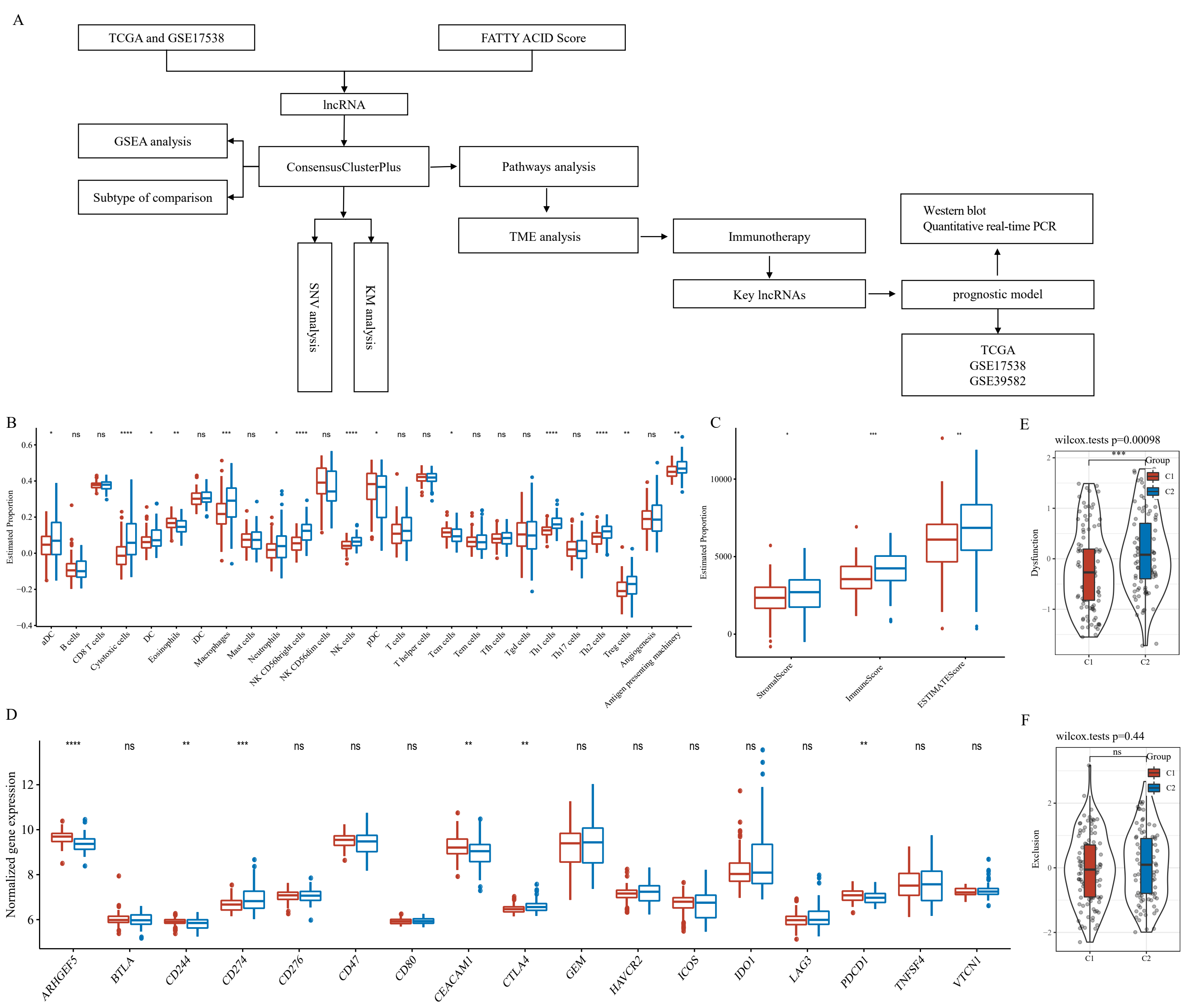

Supplement: Supplementary file 1 — Additional file 1. Supplementary Fig. S1: Workflow and TME features of C1 and C2 subtypes in GSE17538 dataset. A Work flow chart. B Estimated proportions of 24 immune cells. C Stromal score, immune score and ESTIMATE score calculated by ESTIMTAE analysis. D The expression of immune checkpoints in two subtypes. E, F T cell dysfunction and exclusion score calculated by TIDE analysis. Wilcoxon test was conducted. [file 13062_2022_332_MOESM1_ESM.pdf]
