# Supplementary material for: Identification of fatty acid metabolism-related lncRNAs in the prognosis and immune microenvironment of colon adenocarcinoma
Source: Biol Direct. 2022 Jul 28;17:19. doi: 10.1186/s13062-022-00332-y (PMC9331591; doi:10.1186/s13062-022-00332-y)
Supplement: Supplementary file 2 — Additional file 2. Supplementary Table S1: Primer Sequences used in qRT-PCR. [file 13062_2022_332_MOESM2_ESM.docx]

Primer Sequences used in qRT-PCR.

LINC01315-F: 5'-CGGCTTCTCCTCCCAAACATAC-3'

LINC01315-R: 5'-AGCCCATCAGATTTCCGGTAGG-3'

MNX1-AS1-F: 5'-GCCCCGCATTTTCAGATTCAC-3'

MNX1-AS1-R: 5'-AGCCTCGCCATAAAGATCTCCTC-3'

PAXIP1-AS1-F: 5'-CGCGCTCAACAGGTTAATACTACAC-3'

PAXIP1-AS1-R: 5'-TCCGGTCGTGCAGGTGTCTTG-3'

TMEM147-AS1-F: 5'-AACCGGGTGCTTTGCTTCTGTG-3'

TMEM147-AS1-R: 5'-GCCGGAGCCAGGAAGATCAAG-3'

SNHG11-F: 5'-TGCCTTGGGTCTGGAAACTGTTAG-3'

SNHG11-R: 5'-ACCCCCAAACAATCATGAGGAG-3'

β-actin-F 5'-ACCCCGTGCTGCTGACCGAG-3'

β-actin-R 5'-TCCCGGCCAGCCAGGTCCA-3'
