# Supplementary material for: Identification of fatty acid metabolism-related lncRNAs in the prognosis and immune microenvironment of colon adenocarcinoma
Source: Biol Direct. 2022 Jul 28;17:19. doi: 10.1186/s13062-022-00332-y (PMC9331591; doi:10.1186/s13062-022-00332-y)
Supplement: Supplementary file 3 — Additional file 3. Supplementary Fig. S2: Functional analysis of 5 key fatty acid-related lncRNAs. [file 13062_2022_332_MOESM3_ESM.pdf]

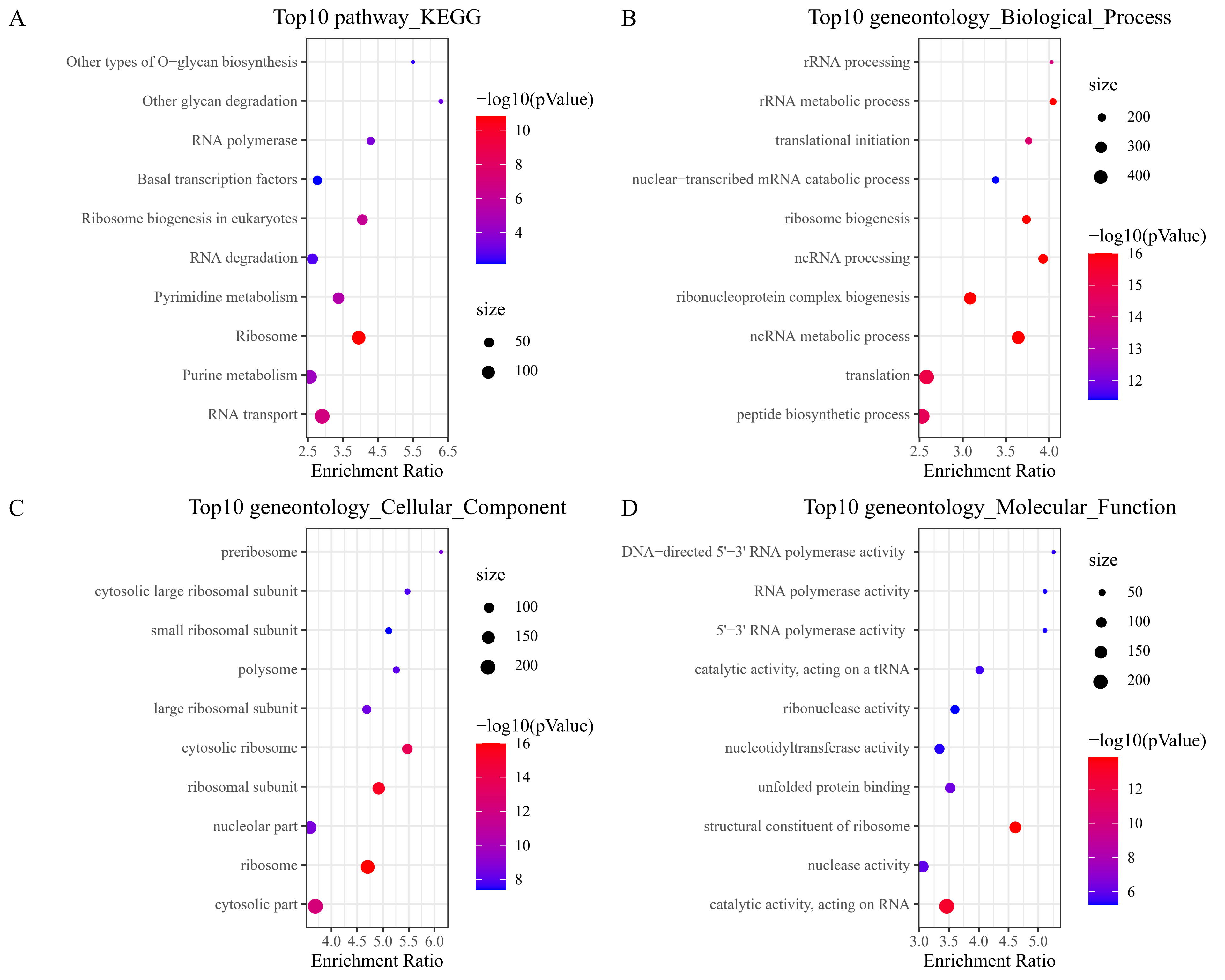
A
Top10 pathway_KEGG
Other types of O−glycan biosynthesis
Other glycan degradation
RNA polymerase
Basal transcription factors
Ribosome biogenesis in eukaryotes
RNA degradation
Pyrimidine metabolism
Ribosome
Purine metabolism
RNA transport
2.5 3.5 4.5 5.5 6.5
Enrichment Ratio
−log10(pValue)
10 8 6 4
size
50 100
B
Top10 geneontology_Biological_Process
rRNA processing
rRNA metabolic process
translational initiation
nuclear−transcribed mRNA catabolic process
ribosome biogenesis
ncRNA processing
ribonucleoprotein complex biogenesis
ncRNA metabolic process
translation
peptide biosynthetic process
2.5 3.0 3.5 4.0
Enrichment Ratio
size
200 300 400
−log10(pValue)
16 15 14 13 12
C
Top10 geneontology_Cellular_Component
preribosome
cytosolic large ribosomal subunit
small ribosomal subunit
polysome
large ribosomal subunit
cytosolic ribosome
ribosomal subunit
nucleolar part
ribosome
cytosolic part
4.0 4.5 5.0 5.5 6.0
Enrichment Ratio
size
100 150 200
−log10(pValue)
16 14 12 10 8
D
Top10 geneontology_Molecular_Function
DNA−directed 5'−3' RNA polymerase activity
RNA polymerase activity
5'−3' RNA polymerase activity
catalytic activity, acting on a tRNA
ribonuclease activity
nucleotidyltransferase activity
unfolded protein binding
structural constituent of ribosome
nuclease activity
catalytic activity, acting on RNA
3.0 3.5 4.0 4.5 5.0
Enrichment Ratio
size
50 100 150 200
−log10(pValue)
12 10 8 6
